# Oxidative stress and toxicity produced by arsenic and chromium in broiler chicks and application of vitamin E and bentonite as ameliorating agents

**DOI:** 10.3389/fvets.2023.1128522

**Published:** 2023-03-08

**Authors:** Javaria Mashkoor, Fatimah A. Al-Saeed, Zhang Guangbin, Abdullah F. Alsayeqh, Shafia Tehseen Gul, Riaz Hussain, Latif Ahmad, Riaz Mustafa, Umar Farooq, Ahrar Khan

**Affiliations:** ^1^Department of Pathology, Faculty of Veterinary Science, University of Agriculture, Faisalabad, Pakistan; ^2^Department of Biology, College of Science, King Khalid University, Abha, Saudi Arabia; ^3^Shandong Vocational Animal Science and Veterinary College, Weifang, China; ^4^Department of Veterinary Medicine, College of Agriculture and Veterinary Medicine, Qassim University, Buraidah, Qassim, Saudi Arabia; ^5^Department of Pathology, Faculty of Veterinary and Animal Sciences, The Islamia University of Bahawalpur, Bahawalpur, Pakistan; ^6^Department of Pre-clinical Studies, Faculty of Veterinary Medicine, Baqai Medical University, Karachi, Pakistan; ^7^University of Agriculture, Faisalabad Sub Campus, Toba Tek Singh, Pakistan

**Keywords:** broiler chicks, arsenic, chromium, toxicity, oxidative stress, vitamin E, bentonite

## Abstract

The present study investigated the adverse effects of arsenic and chromium in broilers and ascertained the role of vitamin E and bentonite in alleviating their harmful effects. For this purpose, we experimented on 180 one-day-old broiler chickens. The feed was administered to broiler chicks of groups 2, 6, 7, 8, and 9 chromium @ (270 mg.kg^−1^ BW). Groups 3, 6, 7, 8, and 9 were administered arsenic @ (50 mg.kg^−1^ BW). Groups 4, 7, and 9 received vitamin E (150 mg.kg^−1^ BW), and groups 5, 8, and 9 received bentonite (5%), respectively. Group 1 was kept in control. All the broiler chicks treated with chromium and arsenic showed a significant (*p* < 0.05) decline in erythrocytic parameters on experimental days 21 and 42. Total proteins decreased significantly, while ALT, AST, urea, and creatinine increased significantly (*p* < 0.05). TAC and CAT decreased significantly (*p* < 0.05), while TOC and MDA concentrations increased significantly (*p* < 0.05) in chromium and arsenic-treated groups on experimental days 21 and 42. Pearson correlation analysis revealed a strong positive correlation between TAC and CAT (Pearson correlation value = 0.961; *p* < 0.001), similarly TOC and MDA positive correlation (Pearson correlation value = 0.920; *p* < 0.001). However, TAC and CAT showed a negative correlation between TOC and MDA. The intensity of gross and microscopic lesions was more in chromium (270 mg.kg^−1^) and arsenic (50 mg.kg^−1^) singly or in combination-treated groups. Thus, broiler chicks treated with chromium plus arsenic exhibited higher gross and microscopic lesion intensity than other treated groups. Fatty degeneration, severe cytoplasmic vacuolar degeneration, and expansion of sinusoidal spaces were the main lesions observed in the liver. Kidneys showed renal epithelial cells necrosis, glomerular shrinkage, and severe cytoplasmic vacuolar degeneration. Co-administration of bentonite along with chromium and arsenic resulted in partial amelioration (group 8) compared to groups 7 and 9, administered arsenic + chromium + vitamin E and arsenic + chromium + vitamin E + bentonite, respectively. It was concluded that arsenic and chromium cause damage not only to haemato-biochemical parameters but also lead to oxidation stress in broilers. Vitamin E and bentonite administration can ameliorate toxicity and oxidative stress produced by arsenic and chromium.

## Introduction

The poultry industry of Pakistan has become an integral part of livestock, contributing 61.89% to agriculture and 14% to GDP. Intensive poultry farming has resulted in poverty alleviation by employing 1.5 million people. With investing > Rs. 750 billion, this industry has been expanding almost 7.5% per annum over the last decade, which has facilitated Pakistan to stand at 11th position among the largest poultry producer in the world and has more than enough room for additional expansion. The poultry industry contributes 38% of the total meat produced in the country (1).

Heavy metals are relentless in the environment and can render bioaccumulation in the food chain. Contamination of drinking water by heavy metals can risk poultry and human health (2). Water pollution due to heavy metals poses a potential risk to human and animal health (3). Wastewater from industrial and domestic sources is responsible for polluting the environment and has become a significant health issue in developing countries (4). Nowadays, heavy metal ions are among water's most toxic inorganic pollutants and have gained ecological significance (5). Heavy metals, including arsenic and chromium, are naturally found in the earth's crust and enter the environment due to natural or anthropogenic pursuits. They have become health hazards worldwide due to their non-biodegradability and bioaccumulation. Biological activities include rock weathering and volcanic eruptions, while anthropogenic activities include mining, ores smelting, and phosphate fertilizer (6).

As a global health issue, arsenic poisoning is influencing millions worldwide because of environmental and occupational disclosure. The fundamental source of arsenic toxicity to the general population is polluted soil, water, and food products (7). Arsenic name is derived from the Greek word “arsenikon” which means potent. Arsenic is the most common and toxic element among the most dangerous xenobiotics listed in the environment (8). Arsenic is found in the environment, e.g., organic and inorganic; their toxicity depends on the form and oxidation state. Arsenite is more toxic than arsenate due to its high affinity to thiol protein groups, while arsenate stops phosphorylation (7).

Chromium (Cr) is a heavy metal notorious as a toxic water pollutant. It is the 21st most abundant element. It naturally exists as mineral chromite and is metallic (9). Organic forms of chromium include chromium picolinate and chromium-enriched yeast. Among inorganic forms, metallic chromium is used to make alloys, i.e., stainless steel. Anti-corrosive property of stainless steel is due to chromium. Trivalent chromium found in water is used in the tannery and in making dyes and paints. Hexavalent chromium is the most treacherous form being utilized in chrome plating and is an important etiology of mutagenesis and cancer (10). Anthropogenic activities, i.e., metallurgy, smelting, electroplating, tannery effluents, and agriculture, has heightened chromium level ahead of the integration ability of the environment. Hence it has become the most toxic element in terrestrial and marine environments (11, 12).

Oxidative stress is a process triggered by inequity between the production and accretion of reactive oxygen species (ROS) in cells and tissues and the capacity of a biological system to cleanse these reactive manufactured items, such as free radicals (13, 14). Free radicals, including superoxide anion, hydroxyl radical, and nitric oxide, usually consist of partially filled orbital having unpaired electrons, which causes the reduction of a variety of organic macromolecules like lipid/protein/carbohydrate (15). Vitamin E renders free radicals inactive due to its ability to donate hydrogen. It is lipid-soluble vitamin located in the cell membrane. It is the best antioxidant available to combat the oxidative stress induced by heavy metals (16).

Among the natural adsorbents available, bentonite is the most widely used clay to eliminate heavy metals from the body. It is used in many ways in broiler feed, such as a binding agent of bacteria/viruses and the pelleting process (17). There are rare studies showing bentonite as an ameliorating agent in arsenic plus chromium intoxicated broiler chicks singly or amalgamation with vitamin E. Thus, this study was planned to know the arsenic plus chromium rendered oxidative stress and further amelioration with bentonite and vitamin E. This study shows how the co-administration of bentonite and vitamin E affects broiler health.

## Materials and methods

### Chemicals

Different chemicals, i.e., Potassium dichromate (K_2_Cr_2_O_7_) and arsenic trioxide (As_2_O_3_), were procured from Merck KGaA, Darmstadt, Germany. We procured vitamin E (α-Tocopherol acetate) from Alpharma Inc., New Jersey, USA. Bentonite was obtained from the Potohar Plateau (Punjab Province, Pakistan), where it is abundantly available.

### Experimental broiler chicks and management

The trial was conducted on 180 one-day-old broiler chicks bought from a regional hatchery and maintained these chicks in wire cages under standard housing and management conditions, i.e., humidity (60–65%) and temperature (24–35°C). We fed broiler chicks a basal diet (chick starter crumbs: 21% total proteins) and plenty of clean water. We administered the Newcastle disease (Nobilis^®^ ND Lasota, Intervet SA (Pty) Ltd) vaccine to these chicks on days 2nd and 23rd. On days 8th and 21st, infectious bursal disease (IBD) [Nobilis Gumboro 228E, Intervet SA (Pty) Ltd], whereas administration of hydropericardium syndrome (BioAngara Plus, Sana Lab) vaccine was on day 19th.

### Experimental design

After 2 days of acclimatization, we randomly divided broiler chicks into nine groups having twenty each. Arsenic, chromium, vitamin E, and bentonite medications began on the 3rd day and remained until 42 days. Potassium dichromate (17), arsenic trioxide (18), and vitamin E (17) were used @ 270, 50, and 150 mg.kg^−1^ BW, respectively. All the treatments were administered daily in the feed. Group 1 served as control. We used 5 % bentonite in the feed (17). Groups 2 and 3 were given chromium and arsenic, respectively. Group 4 received vitamin E while group 5 bentonite. We administered chromium plus arsenic to chicks of Group 6, whereas group 7 received chromium plus arsenic and vitamin E. Group 8 administered chromium plus arsenic and bentonite. Group 9 received chromium + arsenic + vitamin E and bentonite. We assessed each bird in each group for weight weekly, weighed feed intake in each group daily, and then calculated the cumulative average of body weight and feed intake at the end of the experiment.

### Hematobiochemical parameters

Broiler chicks (*n* = 10) were selected randomly from each group and killed humanely on experimental days 21 and 42 to collect blood with anticoagulant (EDTA; 1.0 mg/mL blood) for hematological studies, including total erythrocyte counts, hemoglobin concentration, hematocrit, and total leukocyte counts were determined. Briefly, total erythrocyte and leukocyte counts were figured out following the techniques already described (19) by diluting blood with Natt and Herrick solution and with the aid of a Neubauer counting chamber (Hemocytometer) were counted under a light microscope (20). Using Drabkin's solution, hemoglobin was determined spectrophotometrically (540 nm) through the cyanmethemoglobin method.

### Biochemical studies

The collected blood without anticoagulant was centrifuged (3,000 rpm for 5 min) for serum separation and stored at −20°C. Serum biochemical parameters like total proteins (Cat # 997180), albumin (Cat # 997258), alanine aminotransferase (ALT; Cat # 30254), aspartate aminotransferase (AST; Cat # 30243), urea (Cat # 996060) and creatinine (Cat # 99108) were measured using commercial kits (M/S Canovelles; Barcelona, Spain) with the help of a chemistry analyzer. Globulin was determined by subtracting albumin from total proteins.

### Antioxidant enzymes/parameters

As mentioned under biochemical studies, serum was procured for further studies. We measured total antioxidant capacity (TAC) in the serum samples following the method already mentioned (21). Briefly, by this method, antioxidants present in the sample lessen dark blue-green colored ABTS [2,2'-azino-bis(3-ethylbenzothiazoline-6-sulfonic acid)] radial to colorless ABTS form determined through spectrophotometer at a wavelength between 660 and 670 nm.

The earlier method figured out the catalase (CAT) levels (22). Briefly, free radicals generated, like hydrogen peroxide, respond with molybdate founding a yellowish complex, the intensity of that is measured by spectrophotometer at a wavelength between 352 and 360 nm.

### Total oxidant status

These included total oxidant capacity (TOC) and malondialdehyde (MDA). TOS was figured out using the method already described (23). By this method, ferrous-o-dianisidine is dissolved by oxidants present in the sample forming a ferric ion, which then oxidizes with glycerol by generating color. A spectrophotometer measures concentration at a wavelength of 560 nm.

The MDA was measured according to the earlier procedure (24). MDA oxidizes with deoxyadenosine and deoxyguanosine in DNA, forming complex DNA-MDA and other TBARS abridge with two equivalents of thiobarbituric acid to give a fluorescent red derivative that is assayed with a spectrophotometer at a wavelength of 532 nm.

### Gross and histopathology techniques

The visceral organs, including the lungs, liver, kidneys, and heart, were examined for gross lesions on each killing. Morbid tissues were preserved in 10% buffered formalin and processed for histopathological studies using the routine method of dehydration and embedding in paraffin. For histopathological studies, sections 4–5 μm thick were cut and stained with hematoxylin and eosin (20). Prepared slides were examined under a light microscope. We made the scoring of microscopic lesions based on severity (mild, moderate, and severe).

### Statistical analysis

The data thus gathered were evaluated statistically by utilizing two-factor factorials under a completely randomized design. Group means were equated by Duncan multiple range (DMR) test using Microcomputer Statistical Package (25). Pearson correlations were calculated using Minitab Statistical Software (Minitab Release 13.1). We considered the significance level at *p* < 0.05.

## Results

### Feed intake, body weight, and FCR

All physical parameters like feed consumed, body weight, and FCR decreased significantly (*p* < 0.05) in arsenic plus chromium-administered broiler chicks (group 6) on both experimental days, i.e., 21 and 42. Nevertheless, groups 7, 8, and 9 fed vitamin E and bentonite along with arsenic plus chromium exhibited increased (*p* < 0.05) feed consumption and body weight. In arsenic plus chromium-fed broiler chicks (group 6), there was increased FCR compared to control broiler chicks on investigational days 21 and 42. Groups 7, 8, and 9, treated with bentonite and vitamin E along with arsenic and chromium, exhibited significantly (*p* < 0.05) decreased/improved FCR (Table 1).

**Table 1:** Physical parameters like feed intake, body weight, and feed conversion ratio on experimental days 21 and 42 in broiler chicks fed chromium, arsenic, bentonite, and vitamin E singly or in blends.

**Groups**	**Experimental day 21**			**Experimental day 42**		
	**Feed eaten (g/day)**	**Body weight (g)**	**FCR**	**Feed eaten (g/day)**	**Body weight (g)**	**FCR**
G1	972 ± 14.2a	745 ± 27.5a	1.30	3,397 ± 26.9a	2,074 ± 29.2a	1.63
G2	820 ± 9.3b	515 ± 13.2c	1.59	3,259 ± 15.2b	1,629 ± 15.7c	2.00
G3	821 ± 9.7b	520 ± 12.5c	1.57	3,275 ± 19.9b	1,675 ± 15.9c	1.95
G4	974 ± 13.5a	752 ± 20.7a	1.29	3,392 ± 25.7a	2,079 ± 29.7a	1.63
G5	962 ± 12.9a	741 ± 21.3a	1.29	3,380 ± 23.2a	2,069 ± 29.3a	1.63
G6	795 ± 19.2b	490 ± 10.5c	1.62	3,201 ± 13.7b	1,572 ± 14.2d	2.03
G7	880 ± 11.3c	627 ± 18.5b	1.40	3,265 ± 21.5c	1,859 ± 17.5b	1.75
G8	865 ± 11.7c	617 ± 17.3b	1.40	3,247 ± 14.2c	1,855 ± 17.9b	1.75
G9	969 ± 14.3a	747 ± 23.5a	1.29	3,392 ± 25.3a	2„067 ± 28.3a	1.64

Mean ± SE values with dissimilar alphabets in a column vary significantly (*p* < 0.05). FCR, feed conversion ratio. Chromium, arsenic, and vitamin E were given @ 270, 50, and 150 mg.kg^−1^, respectively, while bentonite was administered @ 5%. G1 = Group 1: Control (negative); G2 = Group 2: Chromium; G3 = Group 3: Arsenic; G4 = Group 4: Vitamin E; G5 = Group 5: Bentonite; G6 = Group 6: Chromium + Arsenic; G7 = Group 7: Chromium + Arsenic + Vitamin E; G8 = Group 8: Chromium + Arsenic + Bentonite; and G9 = Group 9: Chromium + Arsenic + Vitamin + Bentonite.

### Hematological parameters

Displayed a significant (*p* < 0.05) decrease in TEC, hemoglobin, hematocrit, and ESR in groups treated with chromium (group 2), arsenic (group 3), and a combination of arsenic and chromium-treated broiler chicks (group 6) compared with control broiler chicks (group 1) on experimental days 21 and 42 (Tables 2, 3). Vitamin E and bentonite treated groups, along with chromium and arsenic (groups 7, 8, and 9), showed a non-significant (*p* > 0.05) difference compared with control (group 1).

**Table 2:** Hematological parameters including total erythrocyte counts, hemoglobin concentration, hematocrit, and erythrocyte sedimentation rate on day 21 experiment in broiler chicks administered arsenic, chromium, vitamin E, and bentonite alone or in combination.

**Groups**	**TEC (× 10^6^/μL)**	**Hb (g/dL)**	**Hematocrit (%)**	**ESR (mm/h)**
G1	3.39 ± 0.01a	13.0 ± 0.19a	33.6 ± 2.03a	6.97 ± 0.95a
G2	2.75 ± 0.05b	6.35 ± 0.14d	19.5 ± 1.16b	2.97 ± 0.11b
G3	2.89 ± 0.07b	7.57 ± 0.13d	22.5 ± 1.14b	3.21 ± 0.19b
G4	3.37 ± 0.03a	11.3 ± 0.11b	33.1 ± 1.19a	6.92 ± 0.92a
G5	3.32 ± 0.06a	10.1 ± 0.12b	29.5 ± 1.16a	6.72 ± 0.97a
G6	2.65 ± 0.04b	5.15 ± 0.14d	17.3 ± 1.17b	2.85 ± 0.14b
G7	3.19 ± 0.02a	8.97 ± 0.15c	26.3 ± 1.16 b	5.67 ± 0.79a
G8	3.12 ± 0.04a	8.52 ± 0.16c	25.7 ± 1.13b	5.49 ± 0.70a
G9	3.36 ± 0.09a	10.5 ± 0.19d	30.5 ± 1.12a	6.35 ± 0.95a

Mean ± SE values with dissimilar alphabets in a column vary significantly (*p* < 0.05). Chromium, arsenic, and vitamin E were given @ 270, 50, and 150 mg.kg^−1^, respectively, while bentonite was administered @ 5%. G1 = Group 1: Control (negative); G2 = Group 2: Chromium; G3 = Group 3: Arsenic; G4 = Group 4: Vitamin E; G5 = Group 5: Bentonite; G6 = Group 6: Chromium + Arsenic; G7 = Group 7: Chromium + Arsenic + Vitamin E; G8 = Group 8: Chromium + Arsenic + Bentonite; and G9 = Group 9: Chromium + Arsenic + Vitamin + Bentonite. TEC, total erythrocyte counts; Hb, hemoglobin concentration; ESR, erythrocyte sedimentation rate.

**Table 3:** Hematological parameters including total erythrocyte counts, hemoglobin concentration, hematocrit, and erythrocyte sedimentation rate on day 42 experiment in broiler chicks administered arsenic, chromium, vitamin E, and bentonite alone or in combination.

**Groups**	**TEC (× 10^6^/μL)**	**Hb (g/dL)**	**Hematocrit (%)**	**ESR (mm/h)**
G1	3.63 ± 0.07a	11.9 ± 0.12a	32.7 ± 2.04a	7.95 ± 0.92a
G2	2.82 ± 0.09c	6.49 ± 0.16c	20.1 ± 1.13b	3.51 ± 0.14b
G3	2.85 ± 0.02c	7.15 ± 0.11c	23.5 ± 1.12b	3.74 ± 0.17b
G4	3.63 ± 0.02a	9.57 ± 0.17a	33.0 ± 1.14a	7.96 ± 0.93a
G5	3.56 ± 0.03a	9.49 ± 0.15b	30.6 ± 1.16a	7.73 ± 0.99a
G6	2.77 ± 0.06c	5.62 ± 0.11c	19.1 ± 1.19b	3.42 ± 0.12b
G7	3.47 ± 0.04a	9.35 ± 0.12b	27.7 ± 1.16a	6.95 ± 0.74a
G8	3.39 ± 0.03a	9.31 ± 0.13b	25.4 ± 1.17a	6.73 ± 0.73a
G9	3.59 ± 0.01d	10.7 ± 0.12a	31.3 ± 1.11a	7.79 ± 0.95a

The footnote remains the same as that in Table 2.

**Figure 1:**
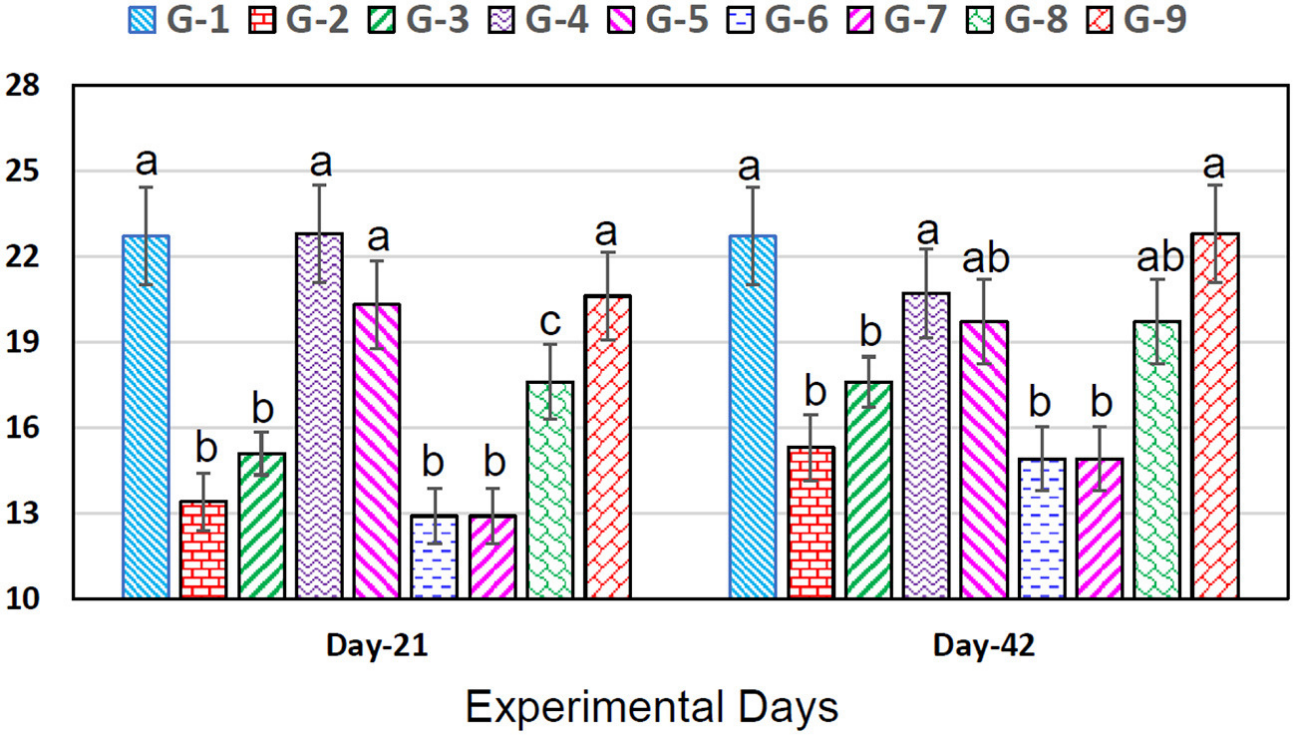
Leukocyte counts (×10^3^/μL) in broiler chicks on 21 and 42 experiment days administered arsenic, chromium, vitamin E, and bentonite alone or in combinations. Bars (mean ± SE) having dissimilar letters under a specific experimental day vary significantly (*p* < 0.05). Chromium, arsenic, and vitamin E were given @ 270, 50, and 150 mg.kg^−1^, respectively, while bentonite was administered @ 5%. G1 = Group 1: Control (negative); G2 = Group 2: Chromium; G3 = Group 3: Arsenic; G4 = Group 4: Vitamin E; G5 = Group 5: Bentonite; G6 = Group 6: Chromium + Arsenic; G7 = Group 7: Chromium + Arsenic + Vitamin E; G8 = Group 8: Chromium + Arsenic + Bentonite; and G9 = Group 9: Chromium + Arsenic + Vitamin E + Bentonite.

Leukocyte counts significantly (*p* < 0.05) decreased in broiler chicks treated with chromium (group 2), arsenic (group 3), chromium + arsenic (group 6), and chromium + arsenic + vitamin E (group 7) at experimental days 21 and 42 (Figure 1). In broiler chicks treated with chromium + arsenic + bentonite (group 8), TLC was higher (*p* < 0.05) on both experimental days than in other 2, 3, 6, and 7 groups but lower (*p* < 0.05) than in control, 4, 5 and 9 groups (Figure 1).

### Biochemical parameters

Significantly (*p* < 0.05) decreased plasma proteins, albumin, and globulin, and urea and creatinine significantly (*p* < 0.05) increased in chromium (group 2), arsenic (group 3), and chromium plus arsenic (group 6) treated broiler chicks compared with control group broiler chicks (Tables 4, 5) at experimental days 21 and 42. Whereas, total proteins, albumin, and globulin showed non-significantly (*p* > 0.05) on experimental days 21 and 42 in broiler chicks treated with chromium and arsenic plus vitamin E or bentonite (groups 7 and 8). Interestingly, broiler chicks in chromium + arsenic + vitamin E + bentonite (group 9) showed identical results in total proteins, albumin, and globulin concentration to control broiler chicks (group 1).

**Table 4:** Biochemical parameters (g/dL) of broiler chicks on day 21 experiment administered arsenic, chromium, vitamin E, and bentonite alone or in combination.

**Groups**	**Total proteins**	**Albumin**	**Globulin**	**Urea**	**Creatinine**
G1	4.12 ± 0.07a	2.55 ± 0.01a	1.54 ± 0.05b	21.3 ± 1.02a	0.40 ± 0.01a
G2	3.45 ± 0.06c	2.14 ± 0.07b	1.31 ± 0.07c	43.5 ± 2.04b	1.17 ± 0.05d
G3	3.49 ± 0.04c	2.16 ± 0.07b	1.33 ± 0.03c	41.7 ± 2.07b	1.10 ± 0.04d
G4	4.14 ± 0.05a	2.54 ± 0.07a	1.36 ± 0.02c	21.0 ± 1.07a	0.40 ± 0.07a
G5	3.93 ± 0.02a	2.17 ± 0.06b	1.76 ± 0.09a	24.1 ± 1.03b	0.51 ± 0.01b
G6	3.37 ± 0.09c	2.10 ± 0.05b	1.27 ± 0.01d	44.5 ± 2.07b	1.23 ± 0.04d
G7	3.65 ± 0.03b	2.29 ± 0.07b	1.36 ± 0.06c	36.9 ± 2.06b	0.69 ± 0.03c
G8	3.61 ± 0.03b	2.22 ± 0.07b	1.39 ± 0.02c	37.7 ± 2.01b	0.75 ± 0.09c
G9	4.07 ± 0.02a	2.30 ± 0.09ab	1.77 ± 0.06a	21.8 ± 0.05a	0.54 ± 0.03b

Values (mean ± SE) with dissimilar letters in a column vary significantly (*p* < 0.05). Chromium, arsenic, and vitamin E were given @ 270, 50, and 150 mg.kg^−1^, respectively, while bentonite was administered @ 5%. G1 = Group 1: Control (negative); G2 = Group 2: Chromium; G3 = Group 3: Arsenic; G4 = Group 4: Vitamin E; G5 = Group 5: Bentonite; G6 = Group 6: Chromium + Arsenic; G7 = Group 7: Chromium + Arsenic + Vitamin E; G8 = Group 8: Chromium + Arsenic + Bentonite; and G9 = Group 9: Chromium + Arsenic + Vitamin E + Bentonite.

**Table 5:** Biochemical parameters (g/dL) of broiler chicks on day 42 experiment administered arsenic, chromium, vitamin E, and bentonite alone or in combination.

**Groups**	**Total proteins**	**Albumin**	**Globulin**	**Creatinine**	**Urea**
G1	4.25 ± 0.09a	2.71 ± 0.07a	1.54 ± 0.02b	0.39 ± 0.09a	23.7 ± 1.07a
G2	3.25 ± 0.01c	1.87 ± 0.06c	1.38 ± 0.02d	1.49 ± 0.06c	38.2 ± 2.07b
G3	3.27 ± 0.07c	1.84 ± 0.02c	1.43 ± 0.07c	1.47 ± 0.09c	37.2 ± 2.07b
G4	4.25 ± 0.03a	2.72 ± 0.02a	1.53 ± 0.07b	0.39 ± 0.01b	23.0 ± 1.09a
G5	4.07 ± 0.09a	2.19 ± 0.07b	1.88 ± 0.01a	0.42 ± 0.06b	23.5 ± 1.05a
G6	3.23 ± 0.01c	1.83 ± 0.09c	1.40 ± 0.03c	1.51 ± 0.03c	39.6 ± 2.06b
G7	3.59 ± 0.05b	2.35 ± 0.07b	1.24 ± 0.09e	0.85 ± 0.07d	33.7 ± 2.06b
G8	3.52 ± 0.02b	1.97 ± 0.07b	1.56 ± 0.06b	0.89 ± 0.07d	35.2 ± 2.06b
G9	4.11 ± 0.07a	2.21 ± 0.03b	1.90 ± 0.05a	0.44 ± 0.02a	25.5 ± 1.01a

The footnote remains the same as that in Table 4.

**Figure 2:**
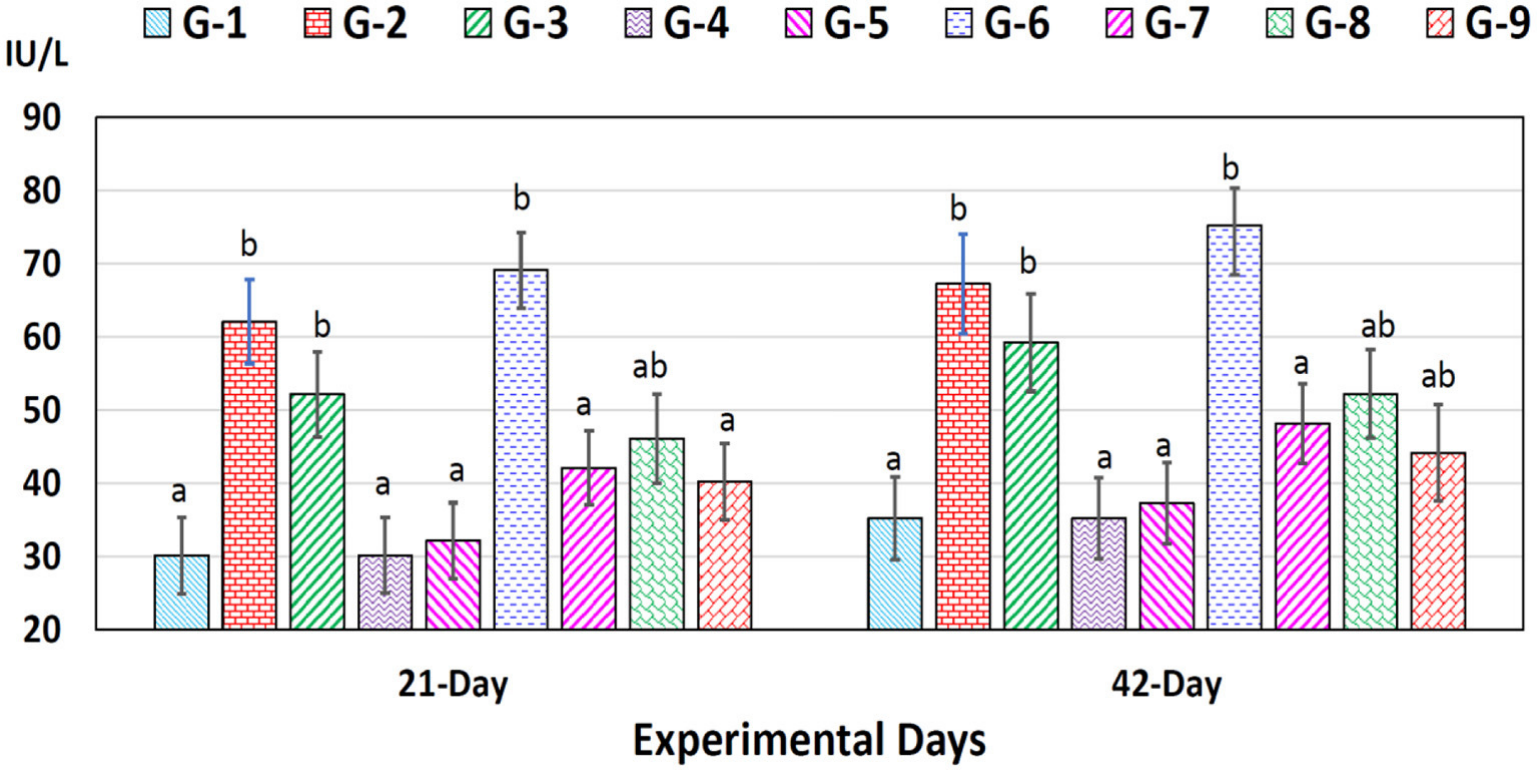
Alanine aminotransferase (ALT) in broiler chicks on 21 and 42 experiment days administered arsenic, chromium, vitamin E, and bentonite alone or in combinations. Bars (mean ± SE) having dissimilar letters under a specific experimental day vary significantly (*p* < 0.05). Chromium, arsenic, and vitamin E were given @ 270, 50, and 150 mg.kg^−1^, respectively, while bentonite was administered @ 5%. G1 = Group 1: Control (negative); G2 = Group 2: Chromium; G3 = Group 3: Arsenic; G4 = Group 4: Vitamin E; G5 = Group 5: Bentonite; G6 = Group 6: Chromium + Arsenic; G7 = Group 7: Chromium + Arsenic + Vitamin E; G8 = Group 8: Chromium + Arsenic + Bentonite; and G9 = Group 9: Chromium + Arsenic + Vitamin E + Bentonite.

ALT concentrations increased significantly (*p* < 0.05) in chromium (group 2), arsenic (group 3), and chromium plus arsenic (group 6) treated broiler chicks compared to control (Figure 2) on experimental days 21 and 42 were observed. Whereas, a non-significant (*p* > 0.05) difference was seen in the concentration of ALT in chromium + arsenic + vitamin E (group 7), chromium + arsenic + bentonite (group 8), and chromium + arsenic + vitamin E + bentonite (group 9) treated broiler chicks with the control group on 21 and 42 trail days (Figure 2).

**Figure 3:**
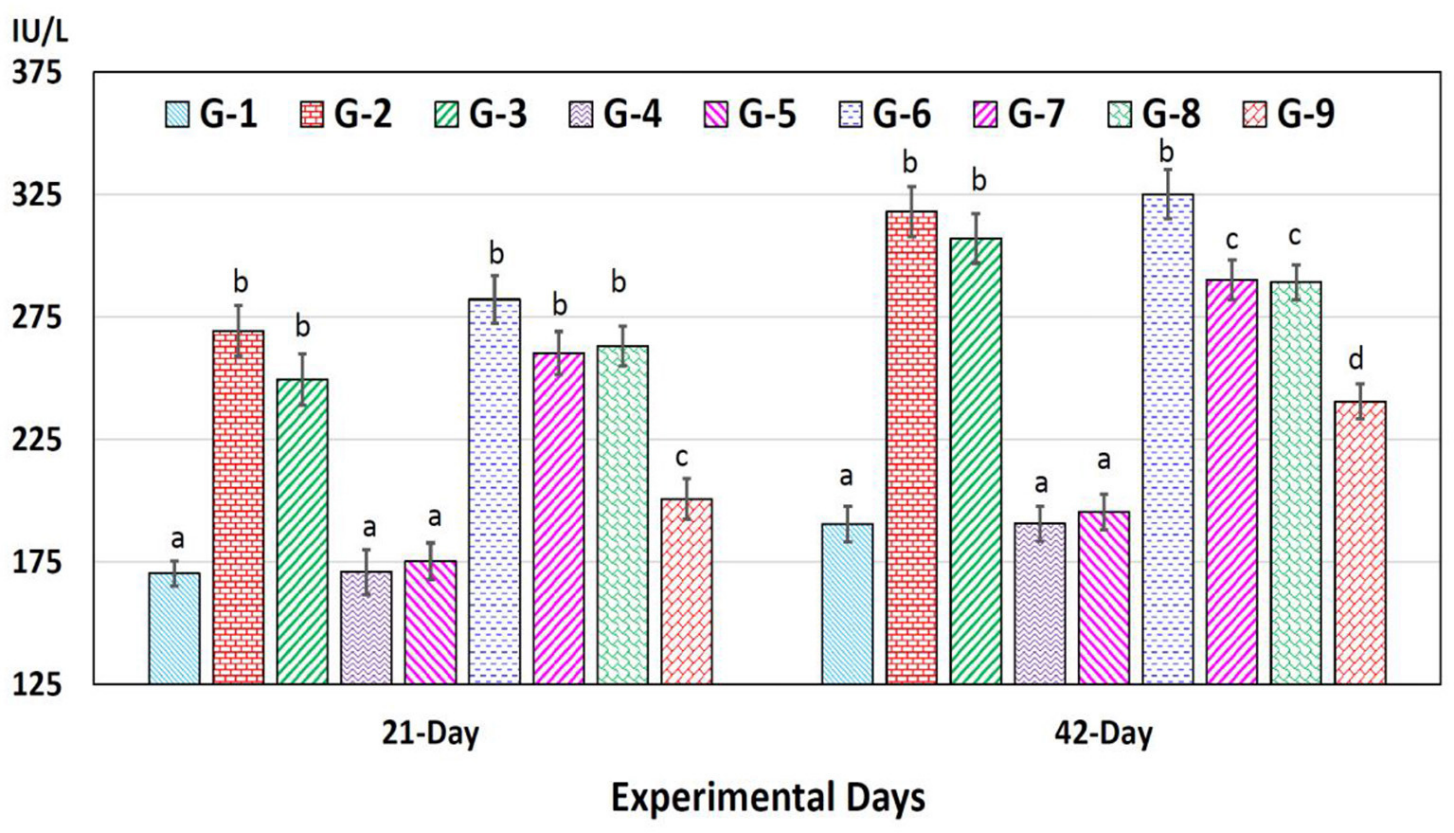
Aspartate aminotransferase (AST) in broiler chicks on 21 and 42 experiment days administered arsenic, chromium, vitamin E, and bentonite alone or in combinations. Bars (mean ± SE) having dissimilar letters under a specific experimental day vary significantly (*p* < 0.05). Chromium, arsenic, and vitamin E were given @ 270, 50, and 150 mg.kg^−1^, respectively, while bentonite was administered @ 5%. G1 = Group 1: Control (negative); G2 = Group 2: Chromium; G3 = Group 3: Arsenic; G4 = Group 4: Vitamin E; G5 = Group 5: Bentonite; G6 = Group 6: Chromium + Arsenic; G7 = Group 7: Chromium + Arsenic + Vitamin E; G8 = Group 8: Chromium + Arsenic + Bentonite; and G9 = Group 9: Chromium + Arsenic + Vitamin E + Bentonite.

AST concentrations increased significantly (*p* < 0.05) in chromium (group 2), arsenic (group 3), chromium plus arsenic (group 6), chromium + arsenic + vitamin E (group 7), chromium + arsenic + bentonite (group 8) and chromium + arsenic + vitamin E + bentonite (group 9) treated broiler chicks compared with the control group at experimentation days 21 and 42 (Figure 3).

### Antioxidant enzymes/parameters

#### Total antioxidant capacity

The TAC of broilers in groups given arsenic, chromium, bentonite, and vitamin E in various amalgamations has been presented in Tables 6, 7. At experimental days 21 and 42, the highest TAC (1.45 ± 0.09 and 0.85 ± 0.02 nmol/L, respectively) was recorded in control broiler chicks (group 1), while significantly (*p* < 0.05) the lowest values (0.82 ± 0.03 and 0.30 ± 0.01 nmol/L, respectively) were observed in broiler chicks treated with chromium and arsenic (group 6). Further analysis revealed that the TAC in broiler chicks of groups 2 and 3 were also significantly (*p* < 0.05) low compared to the control group. Groups administered bentonite and vitamin E along with arsenic and chromium (groups 7–9) showed non-significant (*p* > 0.05) TAC values compared with the control group.

**Table 6:** Antioxidant enzymes/parameters (TAC and CAT) and oxidant enzymes (TOC and MDA) in broiler chicks on day 21 experiment administered arsenic, chromium, vitamin E, and bentonite alone or in combinations.

**Groups**	**TAC (mmol/L)**	**CAT (Kilo U/L)**	**TOP (μMol/L)**	**MDA (nmol/L)**
G1	1.45 ± 0.09a	63.7 ± 3.07a	1.15 ± 0.05a	9.47 ± 0.11f
G2	0.87 ± 0.01b	37.1 ± 1.07c	1.55 ± 0.05b	17.65 ± 0.13a
G3	0.91 ± 0.09b	39.2 ± 1.09c	1.61 ± 0.05b	16.54 ± 0.12b
G4	1.46 ± 0.04a	62.2 ± 3.03a	1.16 ± 0.04a	9.67 ± 0.19f
G5	1.41 ± 0.02a	57.5 ± 2.09a	1.19 ± 0.09a	11.57 ± 0.12d
G6	0.82 ± 0.03b	35.2 ± 0.19c	1.63 ± 0.05b	17.70 ± 0.12a
G7	1.26 ± 0.07a	52.3 ± 2.02a	1.42 ± 0.02c	12.72 ± 0.11c
G8	1.23 ± 0.03a	44.1 ± 1.05b	1.47 ± 0.03c	13.00 ± 0.11c
G9	1.42 ± 0.04a	61.5 ± 3.01a	1.16 ± 0.02a	10.43 ± 0.12e

Mean ± SE values with dissimilar alphabets in a column vary significantly (*p* < 0.05). Chromium, arsenic, and vitamin E were given @ 270, 50, and 150 mg.kg^−1^, respectively, while bentonite was administered @ 5%. G1 = Group 1: Control (negative); G2 = Group 2: Chromium; G3 = Group 3: Arsenic; G4 = Group 4: Vitamin E; G5 = Group 5: Bentonite; G6 = Group 6: Chromium + Arsenic; G7 = Group 7: Chromium + Arsenic + Vitamin E; G8 = Group 8: Chromium + Arsenic + Bentonite; and G9 = Group 9: Chromium + Arsenic + Vitamin + Bentonite. Antioxidant parameters (TAC, total antioxidant capacity; CAT, catalase); oxidant enzymes (TOC, total oxidant capacity; MDA, malondialdehyde).

**Table 7:** Antioxidant enzymes/parameters (TAC and CAT) and oxidant enzymes (TOC and MDA) in broiler chicks on day 42 experiment administered arsenic, chromium, vitamin E, and bentonite alone or in combinations.

**Groups**	**TAC (mmol/L)**	**CAT (Kilo U/L)**	**TOP (μMol/L)**	**MDA (nmol/L)**
G1	0.85 ± 0.02a	73.4 ± 3.01a	1.31 ± 0.01a	10.34 ± 0.15a
G2	0.32 ± 0.02b	49.8 ± 2.09b	1.75 ± 0.02b	18.85 ± 0.19c
G3	0.35 ± 0.09b	50.9 ± 2.02b	1.72 ± 0.02b	17.50 ± 0.19d
G4	0.84 ± 0.03a	73.3 ± 3.01a	1.32 ± 0.01a	10.77 ± 0.12a
G5	0.74 ± 0.09a	70.7 ± 3.09a	1.32 ± 0.02a	11.05 ± 0.11a
G6	0.30 ± 0.01b	47.7 ± 2.07b	1.79 ± 0.02b	19.92 ± 0.14b
G7	0.55 ± 0.05a	61.6 ± 3.04a	1.64 ± 0.01a	15.56 ± 0.19e
G8	0.51 ± 0.07a	59.5 ± 2.03b	1.60 ± 0.09ab	14.50 ± 0.17f
G9	0.82 ± 0.02a	72.6 ± 3.05a	1.32 ± 0.03a	11.67 ± 0.19a

The footnote remains the same as that in Table 6.

Values for CAT of broilers in groups fed chromium, arsenic, bentonite, and vitamin E in various permutations have been presented in Tables 6, 7. A significantly (*p* < 0.05) highest CAT (63.7 ± 3.07 and 73.4 ± 3.01 Kilo U/L, respectively) was recorded in control broiler chicks (group 1). In comparison, the lowest (*p* < 0.05) values (35.2 ± 0.19 and 47.7 ± 2.07 Kilo U/L) were observed on chromium and arsenic-administered broiler chicks (group 6) on experimental days 21 and 42, respectively. Further analysis revealed that the CAT in groups 2 and 3 broiler chicks was also significantly (*p* < 0.05) lower than the control group. Groups treated with bentonite and vitamin E along with arsenic and chromium (groups 7 and 9) showed non-significant (*p* > 0.05) CAT values compared with the control group; however, group 8 showed significantly low (*p* > 0.05) CAT concentration than the control group on experimental days 21 (Table 6) and 42 (Table 7).

#### Total oxidant status

The total oxidant capacity (TOC) of broilers in various groups given chromium, arsenic, bentonite, and vitamin E in different combinations has been presented in Tables 6, 7. On experimental days 21 and 42, significantly (*p* < 0.05) high TAC (1.63 ± 0.05 and 1.79 ± 0.02 μMol/L, respectively) was recorded in chromium and arsenic administration broiler chicks (group 6), while the lowest (*p* < 0.05) values (1.15 ± 0.05 and 1.31 ± 0.01 μMol/L, respectively) was observed in control (group 1). Significantly (*p* < 0.05), high values of TOC were also recorded in chromium (group 2) and arsenic (group 3) treated broiler chicks compared control group. Though significantly (*p* < 0.05) high TOC values were observed in groups treated with bentonite and vitamin E along with arsenic and chromium (groups 7–8) but were significantly (*p* < 0.05) reduced TOC values than values in chromium + arsenic treated broiler chicks (group 6).

Malondialdehyde (MDA) of broilers in groups administered chromium, arsenic, bentonite, and vitamin E in different combinations have been presented in Tables 6, 7. At experimental days 21 and 42, the highest MDA (17.70 ± 0.12 and 19.92 ± 0.14 nmol/L, respectively) was recorded in administered broiler chicks (group 6), while the lowest values (9.47 ± 0.11 and 10.34 ± 0.15 nmol/L, respectively) was observed in control (group 1) with significant (*p* < 0.05) difference. Significantly (*p* < 0.05), higher values of MDA were also recorded in chromium-treated broiler chicks (group 2), followed by arsenic-treated broiler chicks (group 3) compared to the control group. However, higher MDA values were recorded in groups treated with bentonite and vitamin E plus arsenic and chromium (groups 7–9) than in the control group, but MDA values were lower than in chromium and arsenic-treated group (group 6).

#### Pearson correlation

**Figure 4:**
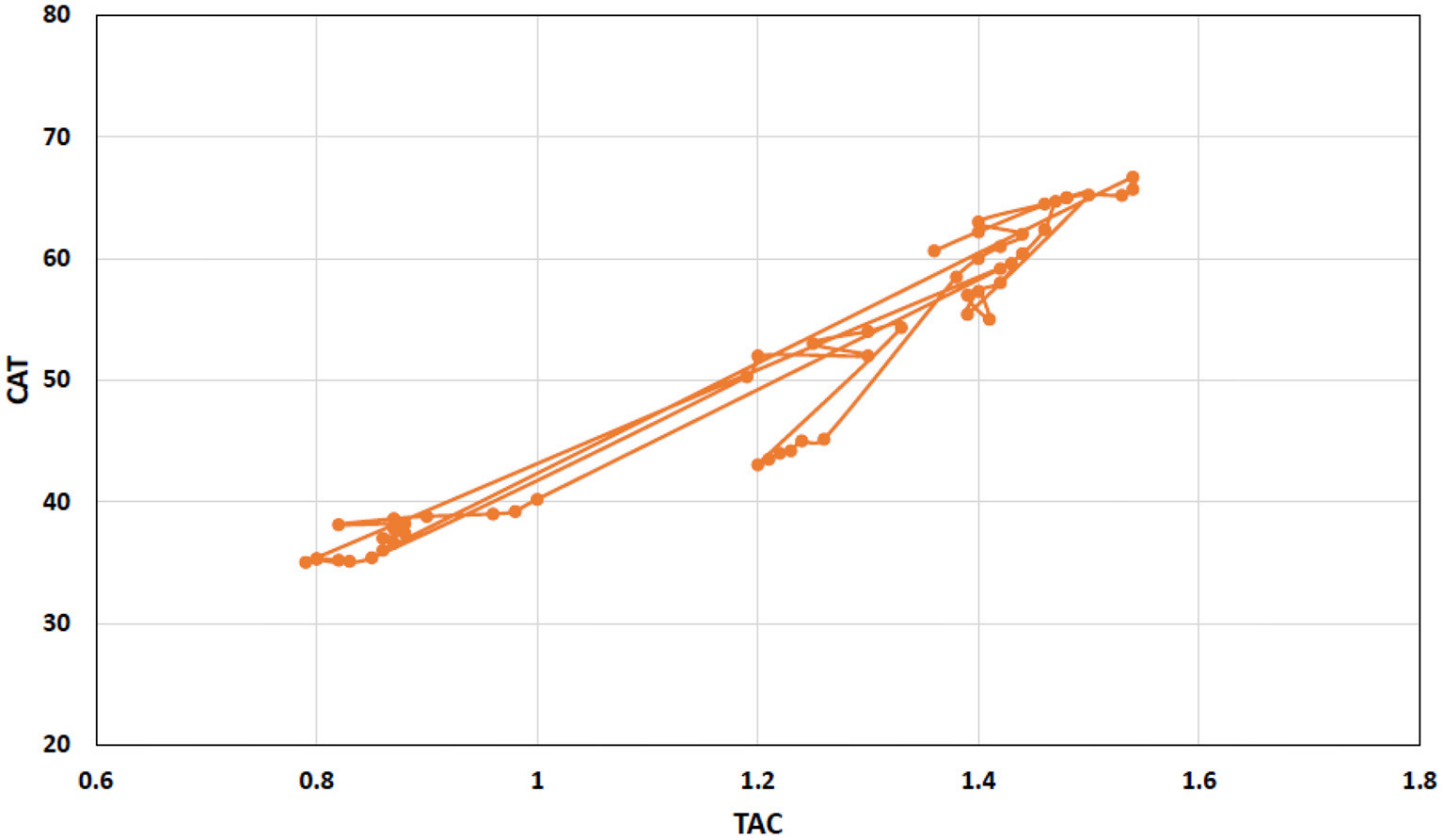
Scattered and point connected graph showing total antioxidant capacity (TAC) positive Pearson correlation with catalase in broiler chicks treated with chromium, arsenic, and their combination along and amelioration with vitamin E and bentonite clay.

**Figure 5:**
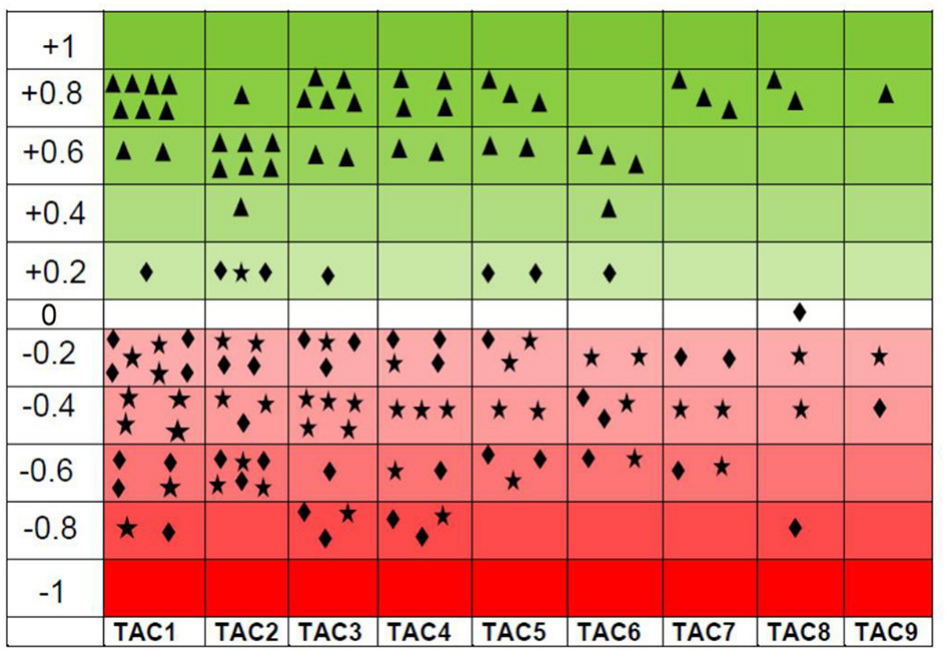
Scattered graph showing total antioxidant capacity (TAC) positive Pearson correlation (green shaded) with catalase (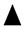) and negative Pearson correlation (red shaded) with oxidant enzymes, i.e., total oxidant capacity (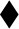) and malondialdehyde (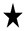) at experimental day 21. There were nine experimental groups. Broiler chicks were treated with chromium and arsenic, and their combination and amelioration of toxic effects with vitamin E and bentonite clay. A positive Pearson correlation has been shown in the green-shaded area from 0 to +1, whereas a negative Pearson correlation has been shown in the red-shaded area from −1 to 0. TAC also has shown a weak positive Pearson correlation with total oxidant capacity and malondialdehyde. A similar trend was observed on experimental day 42. Thus, only the results of experimental day 21 are presented here.

Pearson correlation analysis revealed a strong positive and interconnected correlation between TAC with CAT (Figure 4). We also found a strong positive and significant Pearson correlation between TOC and MDA (Pearson correlation value = 0.920; *p* < 0.001), like that between TAC and CAT (Pearson correlation value = 0.961; *p* < 0.001). However, TAC showed a negative correlation between TOC (Pearson correlation value = −0.075; *p* = 0.590) and MDA (Pearson correlation value = −0.218; *p* = 0.114). Similarly, CAT showed a negative correlation between TOC (Pearson correlation value = −0.021; *p* = 0.882) and MDA (Pearson correlation value = −0.101; *p* = 0.446). Interestingly, with the increase of antioxidant enzymes (TAC and CAT), TOC and MDA decrease and have a negative correlation (Figure 5). TAC1 to TAC6 (group 1: control, group 2: chromium; group 4: vitamin E, group 5: bentonite, and group 6: chromium + arsenic) except TAC3 (group 3: arsenic) showed weak positive Pearson correlation with total oxidant capacity and malondialdehyde (Figure 5).

#### Gross and histopathology

The intensity of gross and microscopic lesions was more in arsenic and chromium singly or in combination-treated groups. It is worth mentioning that broiler chicks of group 6 were treated with chromium (270 mg.kg^−1^) plus arsenic (50 mg.kg^−1^) showed higher intensity of gross and microscopic lesions as compared with other treated groups (Table 8).

**Table 8:** Gross and microscopic lesions recorded in broiler chicks administered arsenic, chromium, bentonite, and vitamin E, alone or in combinations.

**Organ**	**Gross/microscopic**	**Lesions**	**G2 (Cr)**	**G3 (As)**	**G6 (As + Cr)**	**G7 (As + Cr + Vit E)**	**G8 (As + Cr + BN)**	**G9 (As + Cr + Vit E + BN)**
Lungs	Gross	Hemorrhages	++	++	++	+	+	+
		Frothy exudate	++	++	++	+	+	+
	Microscopic	Edema	++	++	+++	+	+	–
		Congestion	++	++	+++	+	+	–
		Emphysema	++	++	+++	+	+	–
Liver	Microscopic	Pyknotic nuclei	++	++	+++	+	++	–
		Expended sinusoidal spaces	+	++	+++	+	+	–
		Cytoplasmic vacuolar degeneration,	++	++	+++	+	++	–
		Leucocytic infiltration	++	++	+++	+	++	–
Kidneys	Gross	Swollen	+	++	+++	+	++	–
	Microscopic	Tubular necrosis	++	++	+++	+	++	–
		Pyknotic nuclei	++	++	+++	+	++	–
		Vacuolation	++	++	+++	+	++	–
		Congestion	++	++	+++	+	++	–
		Glomerular shrinkage	++	++	+++	+	++	–

No lesion was recorded in G1 (Control), G4 (Vit E), and G5 (Bentonite); therefore, they were excluded from this table.

Lesion categorization: No lesion (–), mild (+), moderate (++), and severe (+++). Cr, Chromium; As, Arsenic, Vit E: Vitamin E, BN: Bentonite. Chromium, arsenic, and vitamin E were given @ 270, 50, and 150 mg.kg^−1^, respectively, while bentonite was administered @ 5%. G1 = Group 1: Control (negative); G2 = Group 2: Chromium; G3 = Group 3: Arsenic; G4 = Group 4: Vitamin E; G5 = Group 5: Bentonite; G6 = Group 6: Chromium + Arsenic; G7 = Group 7: Chromium + Arsenic + Vitamin E; G8 = Group 8: Chromium + Arsenic + Bentonite; and G9 = Group 9: Chromium + Arsenic + Vitamin + Bentonite.

Grossly, lungs were normal in size and shape in all treated and control groups except the lungs of groups 2, 3, & 6 (++), and 7, 8 & 9 (+) were hemorrhagic and frothy exudate was seen in the trachea. Microscopically, the major lesions of the lungs, like edema, congestion, and emphysema, were noted in groups 7 & 8 (+), 2 & 3 (++), and 6 (+++). However, mononuclear cell infiltration, hemorrhages, congestion, thickening of alveolar and bronchial septae, and alveolar edema along with emphysema and necrosis were also observed but with intensity. While the lungs of group 1 did not show any microscopic lesions and had well-arranged normal-sized bronchioles and alveoli.

**Figure 6:**
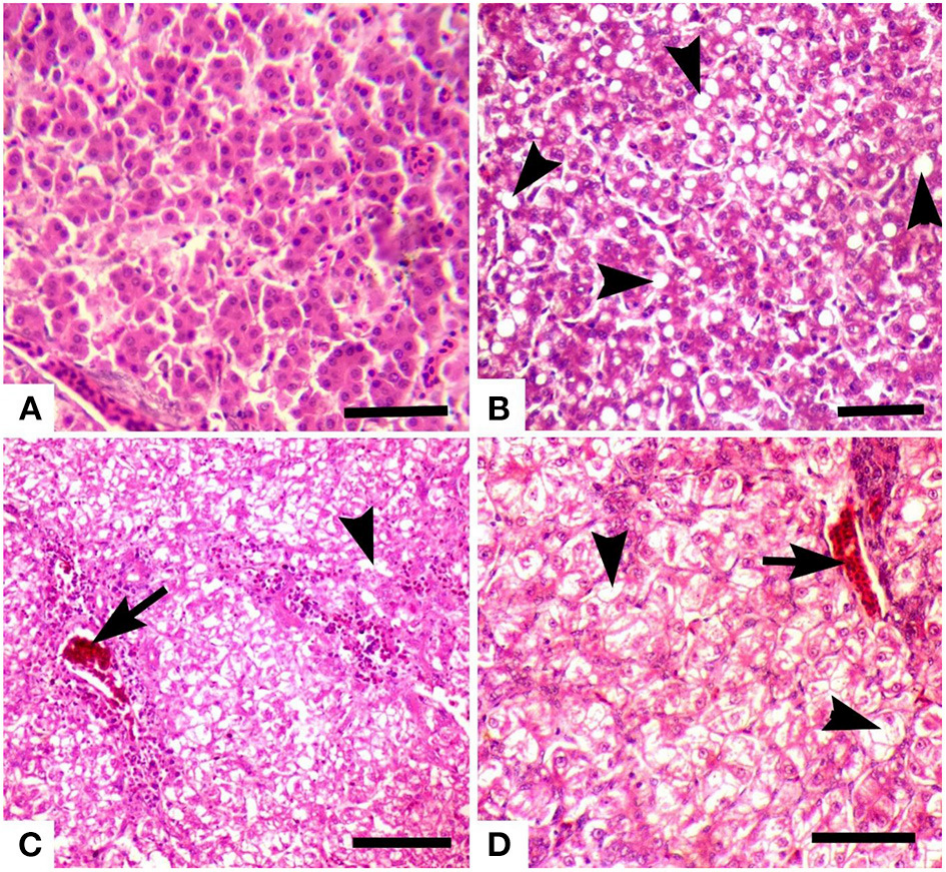
Photomicrograph of the liver of broiler chicks stained with H&E (scale bar = 50): **(A)** control group showing no microscopic lesions, and hepatocytes have well-preserved lobular pattern, **(B)** group 6 (chromium + arsenic treated) showing advanced fatty change in hepatocytes (arrow heads), **(C)** group 2 (chromium treated) showing vacuolar degeneration (arrowheads) and congestion (arrow), and **(D)** group 3 (arsenic treated) showing vacuolar degeneration (arrowheads) and congestion (arrow).

Grossly, the liver was normal in size, shape, and consistency in all groups. Microscopically, pyknosis, condensation of nuclei, along with advanced fatty change in hepatocytes (Figure 6) and mononuclear cells infiltration and separation of cells from the basement membrane in hepatic lobule were observed in groups 7 (+), 2, 3 & 8 (++), and 6 (+++). Advanced fatty degeneration, detachment of cells from the basement membrane, and expansion of sinusoidal spaces were also observed in some treated groups like groups 6 (+++) (Table 8). Nuclear degenerative changes such as karyorrhexis, and karyolysis in broiler chicks treated with chromium + arsenic (group 6) was observed. The liver of group 1 showed no microscopic lesion, and hepatocytes had a well-preserved lobular pattern.

**Figure 7:**
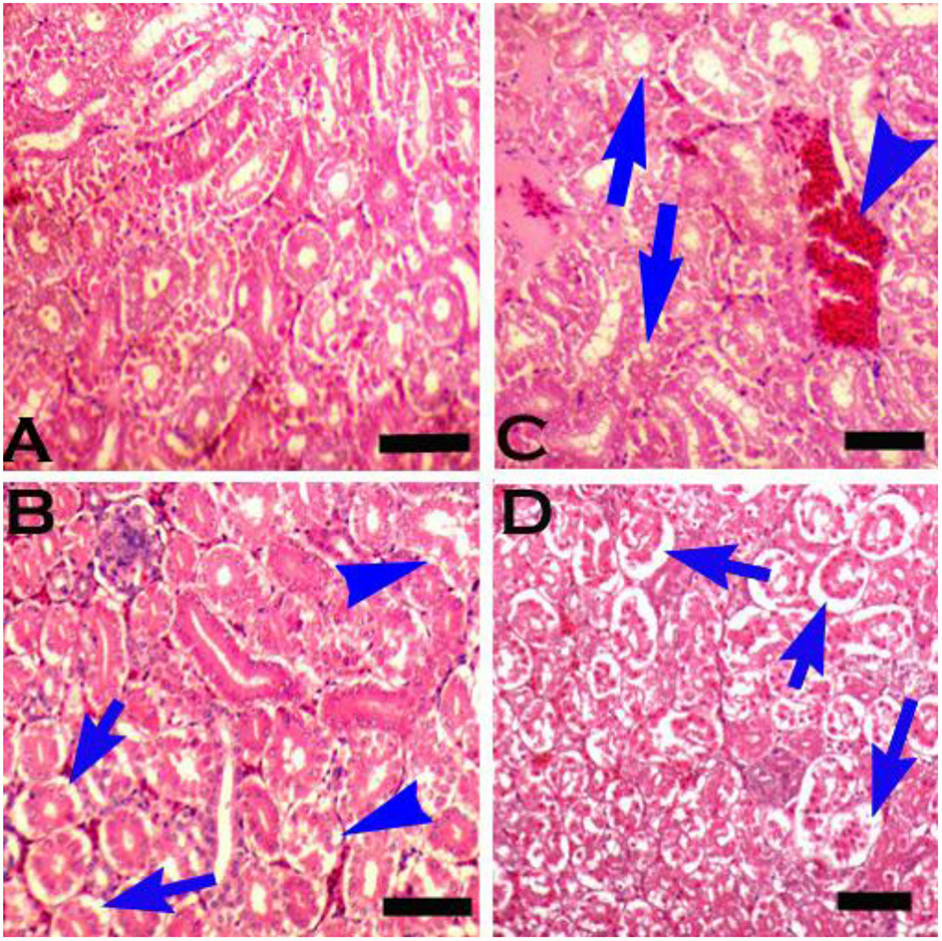
Photomicrograph of the kidneys of broiler chicks stained with H&E (scale bar = 50): **(A)** control group showing normal histology, **(B)** group 2 (chromium treated) showing pyknosis and disintegration of cells from the basement membrane (arrows), and degeneration in the form of vacuolation (arrow heads), **(C)** group 3 (arsenic treated) showing congestion (arrowhead) and degenerative changes in the form of vacuolation (arrows), and **(D)** group 6 (chromium + arsenic treated) showing pyknosis and disintegration of cells from the basement membrane (arrows).

Grossly, kidneys were swollen in groups 2 & 7 (+), 3 & 8 (++), and 6 (+++). Microscopically, renal epithelial cells necrosis characterized by pyknotic nuclei, glomerular shrinkage, increased urinary spaces, and cytoplasmic vacuolar degeneration was observed in a mild form (+) in group 7, moderate (++) in groups 2, 3 & 8, and severe form (+++) in group 6 (Table 8). Degeneration, congestion, disintegration of cells from the basement membrane (Figure 7), karyorrhexis, and karyolysis were also seen in group 6 (+++). There was no microscopic lesion in group 1.

Grossly, the heart was normal in size and shape in all treated groups, i.e., groups 2–9, and did not show microscopic lesions and had fairly well-arranged smooth muscles.

## Discussion

The data of the present study suggested that arsenic and chromium provoke adverse effects not only on hematobiochemical parameters but also on TAC is severely damaged, leading to oxidative stress. The current study showed a significant (*p* < 0.05) decrease in feed intake and body weight in broiler chicks administered with arsenic plus chromium compared with the control group. Earlier reports have also shown a significant reduction in feed intake and body weight in broiler chicks (26) and rats/mice (13) treated with chromium and arsenic, respectively. Decreased feed intake and body weight could be due to the treatment of broiler chicks with heavy metals that could have led changes in liver glycogen and triglyceride along with a disturbance in metabolic enzymes leading to weight loss (27). Decreased feed intake and body weight could also result from metabolic deregulations due to chromium's toxic effects on the liver (11). Another reason could be due to the inhibitory effect of chromium on the specific area of the hypothalamus in the brain regulating feed intake leading to restricted feed intake and ultimately decreased body weight (28).

Erythrocyte indices decreased significantly (*p* < 0.05) in the present study in broiler chicks treated with arsenic and chromium. At the same time, ESR increased significantly in arsenic plus chromium given to broiler chicks compared to the control group in the current experiment. Earlier studies reported decreased hematological parameters because of chromium and arsenic treatment in rats (29) and broilers (30), respectively. A decrease in erythrocytes numbers, hemoglobin concentration, and hematocrit levels indicates an anemic condition, which could be a decrease in the availability of iron for hemoglobin synthesis as a result of heavy metals exposure leading to the development of anemia (31). Another possible reason could be the ability of chromium to cross the red blood cell membrane where it forms DNA protein crosslinks leading to anemia. Anemia could also occur due to the binding of chromium to the β-chain of hemoglobin; thus, hemoglobin would not be available for heme synthesis ultimately anemia develops (28). Chromium taken up by erythrocytes undergoes reduction to the trivalent form with the help of reduced glutathione (32), chromium-hemoglobin complexes, and other intracellular proteins are sufficiently stable to retain chromium for a substantial fraction of the RBC lifetime (33). In this mechanism, arsenic triggers eryptosis either by increasing cytosolic calcium and ceramide concentration or depleting energy (34). The cytopathic effect of arsenic is attributed to its interference with the erythrocytes' energy production pathway leading to interference in ATP production. Arsenic also interferes with mitochondrial enzymes, so there could be no ATP production; ultimately cell lysis occurs (35).

Significantly (*p* < 0.05) decreased leukocyte counts were recorded in arsenic plus chromium-administered broiler chicks compared to control broiler chicks in the present experiment. Earlier studies reported decreased leukocytes following arsenic/chromium administration in rats (11, 36). A decrease in leukocytes could be due to the inhibitory effect of heavy metals on the immune system leading to leukopenia (29). Chromium affects the cortisol level and may be partially liable for its immunostimulatory effects (37). Cortisol influences antibody production and the functions of lymphocytes and other leukocyte populations (38), thus leading to leukopenia. A decrease in leukocytes could result from chromium contact with biological compounds, leading to the peroxidation of these biological complexes present in the cell (11). In effect, some negative changes, such as cell membrane impairment due to the peroxidation of unsaturated fatty acids or inhibition of both mitochondrial trans-membrane potential occur in lymphocytes (39). Still, another possible reason could be due to the capability of chromium to enter the leukocytes and reside there till its life. In this way, it becomes lethal for the leukocytes, thus leading to leukopenia development.

In the current study, ALT and AST increased significantly (*p* < 0.05), while plasma proteins decreased significantly (*p* < 0.05) in arsenic plus chromium-administered broiler chicks compared to the control group. Earlier studies reported increased ALT and AST concentrations in rats (37) and mice (40) for chromium and arsenic, respectively. An increase in the level of ALT and AST could be due to leakage of these enzymes indicating damage to hepatocytes due to heavy metals (chromium/arsenic). Another reason for their increased levels could be a result of the biotransformation of chromium in hepatocytes rendering injury to hepatocytes (41). Heavy metals are known to produce ROS in the body (42). Hepatocytes develop various defensive mechanisms to block ROS consequences. Among the antioxidative enzymes, CAT concentration has been reported to be very high in liver tissue (43). Thus, CAT provides the first line of antioxidative defense enzymatic system, leading to elevated ALT and AST. In the case of arsenic, high serum hepatic enzymes could be due to its binding to the thiol groups of enzymes and proteins of liver cells while arsenic being bio-converted from its toxic (monomethyl arsenic) to less toxic (dimethyl arsenic) metabolites (44). This hepato-cellular membrane damage leads to elevated ALT levels and loss of functions (45).

For the decreased proteins in the present study, there is a possibility that arsenite and trivalent organic (methylated) arsenicals respond with thiols (-SH) in proteins and impede their action (46). A decrease in plasma proteins could also be due to the impairment of podocytes (visceral epithelial cells in Bowman's capsule). The mechanism of podocyte injury could be due to the production of ROS, which is deleterious for podocyte contractile, modulating, and linkage proteins. ROS induces unrepairable damage to podocytes leading to changes in cell integrity, thus affecting the glomerular filtration rate (GFR) and leakage of proteins (47).

Urea and creatinine increased significantly (*p* < 0.05) in arsenic plus chromium-treated broiler chicks compared to the control group in the current study. Earlier reports showed a rise in urea and creatinine in rats (48) for chromium, ducks (49), and goats (50) for arsenic. Urea and creatinine levels could be due to ROS generation, which then causes lipid peroxidation. These lipid droplets sediment in the endothelium of glomeruli, thereby affecting GFR, ultimately damaging the membrane components and leading to necrosis. Therefore, elevated urea and creatinine occur (51). Arsenic has a great affinity to the sulfhydryl group of glomerular filtration membrane; thus, the renal injury could be due to defective GFR (52). After protein metabolism, ammonia is produced. The liver converts it into a less dangerous form as urea which is water soluble and only accumulates in the plasma if the renal system fails to eliminate it from the body (53).

The inequality between the production of ROS and the antioxidant defense system is oxidative stress (13, 14, 54). ROS production is a peculiar feature of heavy metals like arsenic and chromium (46). In this process, mitochondria are the primary organelle affected as the center of cell metabolism. Oxidative stress considerably injures proteins, lipids, and nucleic acids within the mitochondria, resulting in substantial mitochondrial changes in structures and functions (55). Arsenic, a heavy metal, can ruin the anatomy and physiology of mitochondria and yield excess electrons that can convert oxygen (O_2_) into superoxide anion (O${}_{2}^{-}$). Superoxide anions persuade oxidative stress and produce ROS, resulting in lipid peroxidation and MDA formation (56). ROS also persuades DNA breakage, thus generating many molecules of 8-hydroxy-2 deoxyguanosine (8-OHdG) (57). In the meantime, arsenic can trigger the antioxidant defense system and boost the countenance of molecules such as CAT, SOD, GST, and GPx which remove excessive free radicals and peroxides (56). However, if the degree of oxidation exceeds the ability of these antioxidant molecules, then it will reduce the levels of CAT, SOD, GST, and GPx; this is what we have observed in our study in arsenic and chromium-treated broiler chicks.

Total antioxidant capacity is the primary measurement to evaluate the state and potential of oxidative stress. The imbalance between antioxidants and oxidants generates the condition of oxidative stress (56). In the current study, TAC and CAT decreased significantly (*p* < 0.05), while TOC and MDA increased significantly (*p* < 0.05) in chromium and arsenic administration broiler chicks. Heavy metals like chromium and arsenic cause oxidative stress by lessening antioxidant enzymes (TAC, CAT, SOD, GPx, and glutathione reductase) and elevating lipid peroxidation in both target and non-target animals (58). Oxidative stress mediated by ROS is a common denominator in arsenic toxicity (46).

Arsenic and chromium, as individual metals and in combination, affect animals'/birds' health more terribly. The acquaintance with these metals results in the upsurge of oxidative stress that leads to the creation of an uneven number of electrons, triggering the deterioration of proteins, RNA, and DNA and even leading to cell death (56). However, due to the cleansing systems of bare birds/animals, exposure to different toxicants yields rapid and increased formation of ROS. ROS production triggers the lipid peroxidation process, leading to cell membrane damage and the development of TBARS (19). As a result, the increased concentration of oxidative stress indices (TOC and MDA) in the present investigation might be connected to antioxidant enzyme diminution and misbalancing (58).

Depression in CAT concentration after feeding rats (59) chromium and goats (60) for arsenic has been reported. A decrease in CAT levels could be due to the involvement of free radicals. In this situation, SOD is slowed down due to the overproduction of free radicals leading to hyperaccumulation of superoxide because of decreased dismutation of superoxide to hydrogen peroxide; thus, decreased TAC and CAT activity is evident (13).

Levels of TOC and MDA increased significantly (*p* < 0.05) in chromium and arsenic-treated broiler chicks compared to the control group in the current study. Earlier studies reported increased MDA in rats (36) for chromium and cattle (61) for arsenic. MDA is a good marker of lipid peroxidation (62). MDA is well-known toxic metabolite formed by lipid peroxidation due to oxidative stress (63). An increase in MDA level could be due to oxygen free radicals, which further target polyunsaturated fatty acids leading to the production of lipid peroxides that then change membrane fluidity and permeability, ultimately rendering cellular damage (64–66).

Histopathological biomarkers use target organs of toxicity in heavy metal studies, mostly the liver and kidneys (67). The liver and kidneys perform several important functions related to the metabolism and excretion of substances. Thus, lesions in such organs caused by chemical pollutants/heavy metals may negatively affect detoxification and homeostasis (68–70). The present study showed gross and microscopic lesions in chromium (270 mg.kg^−1^) and arsenic (50 mg.kg^−1^) singly or in combination-treated groups. In the liver, the main lesions were fatty degeneration, disintegration of cells from the basement membrane, expended sinusoidal spaces, and cytoplasmic vacuolar degeneration.

In contrast, renal epithelial cell necrosis, glomerular shrinkage, and cytoplasmic vacuolar degeneration were kidney lesions. In White Pekin ducks, inorganic arsenic toxicity has been reported (71). Skin lesions due to chronic arsenic toxicity have been reported (70), as the present study was not chronic, thus, we did not observe these lesions. Chromium toxicity also produces severe lesions in the liver and kidneys (72), as observed in the present study (Table 8).

Several synthetic, as well as natural mixtures have been experienced for the amelioration of arsenic and chromium toxicity (40, 69, 73, 74). Vitamin E, an integral part of the plasma membrane, is an effective antioxidant as it is present at the site of free-radical production; it might counteract the toxic effects of ROS (73). The oral vitamin E and K_2_Cr_2_O_7_ ameliorate all these vicissitudes and ensue in normal hepatic cellular structure and contents (17, 74). Vitamin E is the most effective fat-soluble non-enzymatic antioxidant, which safeguards the cell membrane from radical-induced peroxidation, rouses the initiation of antioxidant enzymes, and lessens the concentration of oxidative stress produced by heavy metal-induced toxicity (75, 76).

It has been explained earlier that vitamin E allows free radicals to non-concrete a hydrogen atom from the antioxidant molecule rather than from polyunsaturated fatty acids, thus breaking the chain of free radical regeneration, thus resultant antioxidant radical being a comparatively unreactive species (40). Vitamin E, due to its hepatoprotective properties, has got wide attention, which is principally due to its capability to lessen the tempted oxidative stress in various tissues by reducing MDA levels, restoring the levels of CAT, SOD, GSH, and the recovery of impaired hepatocytes (74).

Vitamin E administration improves various hemato-biochemical and oxidative stress parameters in arsenic plus chromium-administered broiler chicks compared to the control group in the present study. Oxidative stress results from a disproportion in free radical generation and antioxidant production. A possible mechanism of vitamin E-induced restoration of antioxidant enzyme levels is breaking the chain reaction initiated by free radicals (77). It repairs the oxidizing radicals responsible for the chain elongation by autoxidation. Its antioxidant activity could also be due to its ability to restore the cell membrane to normal by interacting with the unsaturated fatty acid chain (77). Moreover, vitamin E is an important component of the cytoplasm and cell membrane (75). Vitamin E is efficient only in protecting the outer cellular layer of cells from oxidation stress, although very low concentration still prevents lipids and proteins from oxidation (13, 36). Mechanism regarding chain-breaking antioxidants states that SOD, GPx, and CAT are responsible for removing super oxides and peroxides before metal catalyzes them to generate free radicals (78). However, some free radicals escape the protective mechanism of the enzymes, and a peroxidative chain reaction occurs. Here chain breaker antioxidants, i.e., vitamin E plays an important role by donating electrons, thus limiting this cascade of deterioration (14).

Bentonite is a clay mineral collection of fine particles with high opening volume and particular active sites (17, 79). Bentonite is a widely used porous adsorbent aluminum phyllosilicate clay with high adsorption capability, chemical and mechanical solidity, and unique inter-lamellar structural properties (80, 81). The metal ion belongings, preliminary concentration, adsorbent quantity, and operational circumstances (contacting time, pH, temperature, etc.) are the most important factors for the successful application of raw and altered bentonite (79). Metals like arsenic, chromium etc., interfere with various body processes and are toxic to many organs and tissues (82), thus leading to the generation of ROS, consequently creating oxidative stress in the body (46). Bentonite absorbs these toxic substances and acts as an ameliorating agent (80, 83). In the present study, bentonite administration improved arsenic and chromium-treated broiler chicks compared to the control group. Partial amelioration by bentonite could be due to its ability to prevent heavy metal absorption by forming inert, stable, and insoluble complexes with toxic substances (84, 85). Another proposed mechanism of partial amelioration of bentonite could be due to its adhesive ability and absorptive nature by which it attaches to toxic substances occurs, rendering their absorption lessened (17, 86, 87).

## Conclusion

The data suggested that arsenic and chromium aggravate adverse effects not only on hematobiochemical parameters but also lowering total antioxidants, thus enhancing oxidative stress. All the broiler chicks treated with chromium and arsenic showed a significant (*p* < 0.05) decline in erythrocytic parameters. Total proteins decreased significantly, while ALT, AST, urea, and creatinine increased significantly (*p* < 0.05). TAC and CAT decreased significantly (*p* < 0.05), while TOC and MDA concentrations increased significantly (*p* < 0.05) in chromium and arsenic-treated groups. There was a strong positive correlation between TAC and CAT (Pearson correlation value = 0.961; *p* < 0.001), with similar TOC and MDA positive correlation (Pearson correlation value = 0.920; *p* < 0.001). However, TAC and CAT showed a negative correlation between TOC and MDA. The intensity of gross and microscopic lesions was more in chromium (270 mg.kg^−1^) and arsenic (50 mg.kg^−1^) singly or in combination-treated groups. Lungs of arsenic and chromium treated broiler chicks were hemorrhagic and had frothy exudate in the trachea and microscopically, lungs edema, congestion, thickening of alveolar and bronchial septae, and necrosis were observed. Heart was also normal in size and texture in treated broiler chicks. In the liver, fatty degeneration, detachment of cells from the basement membrane, severe cytoplasmic vacuolar degeneration, and expansion of sinusoidal spaces, while in kidneys, renal epithelial cells necrosis, glomerular shrinkage, and severe cytoplasmic vacuolar degeneration were the main lesions. Co-administered bentonite along with chromium and arsenic resulted in partial amelioration compared to groups administered arsenic plus chromium plus vitamin E and arsenic plus chromium plus vitamin E plus bentonite, respectively. Vitamin E and bentonite administration can ameliorate toxicity and oxidative stress produced by arsenic and chromium.

## Data availability statement

The original contributions presented in the study are included in the article/supplementary material, further inquiries can be directed to the corresponding author.

## Ethics statement

The animal study was reviewed and approved by Bioethics Committee, University of Agriculture, Faisalabad, Pakistan.

## Author contributions

AK tailored the research project and managed the experiment. JM executed the project with the help of STG, RH, LA, RM, and UF. JM, STG, and RH carried out laboratory work and data analysis. AK, RH, FAAS, and AFA interpreted the data. The manuscript was written by AK and checked by ZG. All authors approved the final version of the manuscript.
